# A public health development intervention between acceptability and feasibility: descriptive-interpretive discussion of an example

**DOI:** 10.11604/pamj.2024.47.153.42486

**Published:** 2024-04-02

**Authors:** André Nana Yakam, Jürgen Noeske, Irène Adeline Goupeyou Wandji

**Affiliations:** 1 University of Douala, Faculty of Economics and Applied Management, Douala, Cameroon,; 2 Deutsche Gesellschaft für Internationale Zusammenarbeit, Yaounde, Cameroon,; 3 Littoral Regional Delegation of Public Health, Regional Technical Unit of the National Tuberculosis Program, Douala, Cameroon

**Keywords:** Child contact, preventive treatment of tuberculosis, 3RH, acceptance, Cameroon

## Abstract

**Introduction:**

the National Tuberculosis Control Program (NTP) in Cameroon participated between 2016 and 2018 in a multi-country operational study of the Union against Tuberculosis and Lung Disease (The UNION) aiming at demonstrating the efficiency and feasibility of systematic tuberculosis preventive treatment (TPT) with 3 months of an isoniazid/rifampicin (3RH) combination in under-five child contacts of bacilliferous TB patients. Cameroon was one of the participating countries of the study. Despite the promising results communicated following this study, the coverage of TPT with 3RH in Cameroon remains low. We explored the intervention under aspects of acceptability and perceived feasibility.

**Methods:**

key participants and stakeholders in this descriptive interpretative study in Cameroon were interviewed in five focus groups or individually (31 individuals). The Focus Group Discussion (FGD) and interview transcripts were analysed for different components of acceptability using a theoretical framework and the results discussed confronting them with the main objective of the study, i.e. demonstrating feasibility.

**Results:**

the children's parents expressed overall positive feelings about and acceptance of the intervention, emphasizing the unexpected empathy shown by the health staff. The involved field staff, too, showed unreserved acceptance. On the other hand, managers at the intermediate and central levels showed scepticism as to the process of initiation of the study as well as to its feasibility in the given context, neglecting aspects of resources necessary for a scaling-up and of prioritisation.

**Conclusion:**

the adoption of a public health strategy, also internationally recognized as an effective and efficient intervention, requires more than the demonstration of its acceptability or feasibility during the term of a showcase project introduced by an external development partner. Adoption is conditioned by adoption and circumspect planning involving at each stage the stakeholders on all levels of the program.

## Introduction

The ´Stop TB Strategy´ calls for a 90% reduction in the incidence of tuberculosis (TB) by 2035 by promoting early detection, treatment, and prevention for all [1]. About 11% of tuberculosis (TB) cases are believed to be children, a risk group especially at young ages. Long-time neglected as a target of national tuberculosis programs (NTPs), however, the prevention of paediatric TB has become an important part of the elimination strategy [2-5]. However, the gap between this advocated strategy and the implementation on the ground remains considerable. According to the World Health Organization (WHO) World Tuberculosis Report (2022), only 55% of child contacts under 5 years of age TB patients received tuberculosis preventive treatment (TPT) [6].

Since 2012, the guidelines of the Cameroonian NTP insist on the systematic investigation of child contacts aged 0-5 years of patients with bacilliferous TB (TBB+) [7]. Between January 2016 and December 2018, the International Union against Tuberculosis and Lung Disease (The UNION) conducted an operational study in four countries, including Cameroon, with the “...objectives (...) to demonstrate the feasibility of contact investigation and preventive therapy with standard and shorter regimens (rifampicin-isoniazid (RH 75/50) during 3 months) for children under 5 years of age in contact with bacteriologically confirmed pulmonary TB patients and to document their effectiveness“. The protocol and results of this study *Transmission Intégrée de la Tuberculose Infantile* (*TITI*), published elsewhere, concluded that (i) The home visit is essential to allow registration and the first clinical evaluation of child contacts of TBB+ patients; and that (ii) TPT of children under 5 in contact with 3RH is feasible, well tolerated and effective in preventing active TB under programmatic conditions [8]. In the same vein, the Elizabeth Glaser Paediatric AIDS Foundation (EGPAF) launched in 2017 in nine African countries, including Cameroon, a project also aimed at increasing TPT coverage [9]. Nevertheless, in Cameroon in 2022 less than 25% of child contacts of TBB+ patients received TPT, moreover, with the prolonged treatment for 6 (six) months with isoniazid.

The effectiveness of standardized short-course TPT for under-five children is endorsed by WHO already since 2018 in corresponding guidelines and a handbook [10,11]. The *TITI* intervention as by the way the EGPAF intervention aimed, too, at demonstrating feasibility without, however, defining it further. The unspoken intention of the *TITI* study, a development intervention, was apparently to impact the NTP´s strategic choices by demonstrating the feasibility of an effective and highly recommended strategic intervention. The implicit ...’theory of change´ can be supposed the trust in the persuasive power of the study´s promising results able to influence the NTP´s strategic orientations towards TPT with 3RH for child contacts of TBB+. Acceptability by recipients´ and by ´programme deliverers´ is one of the general areas of focus addressed by feasibility evaluations [12]. Our study has two objectives, a) to explore retrospectively the acceptability of the *TITI* study by the target groups of the intervention, i.e. parents of targeted children and implicated nurses; and b) to evaluate the acceptability and perceived feasibility of the *TITI* study by the NTP programme staff. Exploration and evaluation are presented in two sequential steps.

## Methods

**Design and setting:** this retrospective, sequential, qualitative, and descriptive-interpretive study was conducted during the period of October to December 2022 in the four Diagnosis and Treatment Centres (DTC), targeted by the *TITI* intervention in Douala, Cameroon, and in the offices of the interviewed NTP managers (Douala, Yaounde). FGDs and interviews at the regional level were organized and held by authors (ANY and/or AGW and/or JN), and the interview at the central level was done by author ANY, all three have proven experiences with programme monitoring and evaluation and is known by the participants and interview partners as resource persons of the NTP.

### Step 1: exploration of acceptability by recipients

**Participants:** the study population included parents of children recruited into the study. Four Focus Group Discussions (FGD) with parents of children targeted by the study were held, one in each of the four participating DTCs. Candidate-parents were contacted by telephone or, when still under TB care control, face-to-face, and purposely recruited for the FGDs until the desired number was obtained (5-6 participants). About three in five approached parents refused participating in the FGD, the main reasons given were practical constraints or lack of interest.

**Generation and analysis of data:** based on the theoretical acceptability framework developed by Sekhon *et al*. and adapted by Wademan *et al*. a thematic check-list with domains of acceptability (usability, receptivity, user-health system interface) were used to guide the FGDs [13,14]. A digital voice recorder (DVR) was used as a data collection tool. FGDs were recorded and transcribed verbatim with verification of the transcripts; during FGDs notes were taken by the facilitator. Each FGD had a duration of 90 to 120 minutes. Analysis of the FGD discussions was done by ´guided´ content analysis: the participants´ contributions were analysed and coded for the seven components proposed by the theoretical framework (Sekhon): (i) Affective attitude; (ii) burden; (iii) ethicality; (iv) understanding of the intervention; (v) opportunity costs; (vi) effectiveness; and (vii) self-efficacy [12]. The items of analytical interest according to the framework were firstly coded and mapped by the authors individually (ANY, JN, IAGW) and then, after consensus, definitely coded. The part of the contributions not fitting in the conceptual mapping was scrutinized for additional nuances of the concept of acceptability. Given the circumstances (distance of parents´ homes, logistic means available, minimal possible socio-psychological and economic consequences for the participants contributing to the item discussed) transcripts were not returned to the participants for comments or corrections.

### Step 2: evaluation of the *TITI* study´s acceptability and perceived feasibility by NTP staff

**Participants:** the study population included the five female nurses directly involved in the *TITI* study, three regional NTP staff members purposely identified in Douala, the location of the study, and two key actors at the central level, among them the present (former deputy) coordinator of the NTP at the central level. A Focus Group Discussion (FGD) was held with the five nurses, four among them in charge of the execution of the intervention (identification of the target population, home visit, recruitment, clinical and administrative follow-up) and one nurse primarily charged with the monitoring of activities. All five nurses consented to participate and participated actually. In-depth interviews were held individually with the NTP staff members in their respective offices.

**Generation and analysis of data:** the thematic check-list guiding FGDs and interviews exploring acceptability and perceived feasibility of the TITI study and evaluating the study as a public health intervention comprised the above-listed acceptability domains, but included adapted evaluation domains for public health interventions. The latter were adapted from the OECD evaluation criteria for the evaluation of development projects (EvalNet) i.e. relevance and coherence, effectiveness, efficiency, impact and sustainability [15]. Procedures with regard to recording, transcription, and field-notes taking were similar to step 1 described above. The FGD had a duration of about 120 minutes, and interviews of about 90 minutes. Analysis of the FGD discussion as well as of the interviews followed the same procedure as described in step 1 as a ´guided´ content analysis. Concerning acceptability, the aspects of ´burden´, ´ethicality´, ´understanding of the intervention´, ´opportunity costs´ were explored and coded for, ´effectiveness´ being shared as an overlapping category with the development intervention evaluation criteria. Analysing, coding, and mapping were done by the authors for acceptability and the intervention evaluation first individually, and then finally consensually coded. The part of the contributions not fitting in the conceptual mapping was scrutinized for additional nuances of the concept of acceptability. Due to logistic obstacles, transcripts were not returned to the participants for comments or corrections. All methods were checked by COREQ guidelines. (COREQ criteria) [16].

**Ethics and consent:** the Institutional Human Health Research Ethics Committee of the University of Douala (Cameroon) approved the study (n°2812/IEC-UD/07/2021/T). FGD participants were compensated with a financial contribution to their travel costs. Informed consent was obtained from adult participants and parents of the child participants prior to data collection. All data collected was treated confidentially and used only within the frame of the study; the contributions of actors have been anonymized.

## Results

**Acceptability by the recipients:** between October 15 and December 28, 2021, a total of 22 parents participated in four focus group discussions; none of the contacted and assenting parents renounced participating. The number and characteristics of parents participating in FGD and interviews are listed in Table 1. Overall, parents of children recruited into the study expressed positive feelings about the intervention and welcomed it for a variety of reasons (Annex 1). Of importance to them was the unusual and unexpected attention to their and their children´s health problems by the health personnel, perceived as ´patient-centered care´. Parents became familiar with and integrated into their value system the notion of (TB) disease prevention. They relied on the proposed measures, confident about their effectiveness; and they were willing to bear certain burdens such as time constraints or other constraints related to caregiving. One participant of the FGDs, among the 7% (83/1186) of parents of index cases reported having refused to participate in the intervention, strongly expressed doubts about the effectiveness of the intervention. The wording of his refusal leaves open the underlying motives. Skepticism and distrust of preventive measures in general, overcompensation with reaction formation (according to psychoanalysis) thus turning prevention into an intervention favouring disease instead, refusal to participate in a 'medical experiment', apprehension that additional charges might occur confronted with health services known to 'ransom' patients, all together may have played a role.

**Table 1:** number and characteristics of participants of focus group discussions exploring acceptability by recipients of a study aiming to provide tuberculosis prophylactic treatment (TPT) to children less than 5 years old in selected health facilities in Douala, Cameroon (2023)

No. of order	DTC participants	Number	Gender	Age - variance
1	Hôpital de Laquintinie	6	2 males/ 4 females	26-45
2	Mboppi Baptist Hospital	6	1 male / 5 females	30-42
3	New Bell, District Hospital	5	5 females	25-45
4	Cité des Palmiers, District Hospital	5	1 male / 4 females	27-47

DTC: diagnosis and treatment centres

**Acceptance and feasibility as perceived by NTP health staff at the regional level:** the health personnel involved accepted and supported the intervention wholeheartedly during the time of its execution. It was emphasized that the duration of the habitual TPT had been reduced: *“before this project, we gave Isoniazid (INH) to children for six months...it was a burden even for the family, even for the children”*. Satisfactory was also the fact of participating systematically in a prevention intervention: *“It was a pleasure for me to prevent tuberculosis in the participating children (being) as contacts at risk for the disease”*. The staff, partly released from work for the study, reimbursed for transport and communication costs, and incentivized for the additional time spent in consultation and during home visits, did not feel their workload increased beyond the tolerable. Participation in the intervention was seen more as an intellectually and professionally enriching opportunity: *"I learned a lot"* or *"I had the opportunity to go into the field, I had never done it before"*or, again, *"now, when the TITI project arrived, it made us understand that the management of tuberculosis in children under five was a necessity"*. The health personnel wanted the intervention to be scaled up nationally: *"we have to continue, there are our results, the families are happy, the nurses are happy, the children who have taken (TPT) are happy, it (i.e. the intervention as a programmatic strategy) should be adopted”*. But, simultaneously clear-cut doubts are expressed about the feasibility of programmatic scaling up in the prevailing situation. Missing resources are the main issue evoked: *"we could continue, but with a lot of funding for field visits, communication costs, radio, transport costs for check-up consultations for children”*.

**Acceptance and feasibility as perceived by the central NTP management:** number and characteristics of NTP management resource persons interviewed are listed in Table 2. When contacted by the funding agency, the acting NTP coordinator accepted, seemingly, without reservation the intervention, gave administrative clearance, and indicated a setting as to where to conduct the study. As it appears, this process happened in a somewhat cocooned way. Without doubting in principal effectiveness or operational merits of TPT with 3RH for child contacts of TBB+ patients, one of the key actors of the NTP (having inherited the administrative clearance together with the study from the former coordinator) stated: *“we (i.e. central NTP management staff) were not involved in the implementation of the project”*. To ensure continuity and scaling up, *“...(the) NTP monitoring and evaluation team (should have) fully participated in the monitoring and implementation of activities”*. Or as stated by another central level actor: *“the information was not well shared...The means available within the NTP cannot support the additional activities of the project because the extension of the project requires significant funding to support all the activities. The project cannot continue after the UNION because government cannot support it”*, NTP actors consider the project to have been carried out in a vacuum in a limited number of CDTs with benefits limited to the target populations of the intervention: *“although it also probably contributed to the prevention and care of tuberculosis among children and to the sensitization and training of personnel”*, he congruously concludes that *“the intervention has remained without perceptible results for the country”*. Questioned about the prospects for scaling up, the answer is unambiguous, joining the skepticism of medical personnel and best resumed by an interview partner at a regional level: *“so that's the problem, I'm not sure, because you need the staff, you need the equipment even in the laboratory, the staff who are in the DTCs, it's a staff who is already affected to perform certain tasks. So for us to bring in a new activity, we need additional staff, additional equipment, as well as the means”*.

**Table 2:** number and characteristics of participants of a focus group discussion and interviewed managers at the regional/central level of the NTP recruited for the assessment of acceptability and perceived feasibility of a study aiming at promoting tuberculosis prophylactic treatment (TPT) to children less than 5 years old in selected health facilities in Douala, Cameroon (2023)

No. of order	Place of FGD/interview	No. of participants	Gender distribution	Age - variance
1	Regional Delegation of Public Health	5	5 females	29-39
2	Regional Delegation of Public Health	3	3 females	38-57
3	NTP Central Office	2	2 males	55-60

FGD: Focus Group Discussion; NTP: National Tuberculosis Control Program

## Discussion

Development interventions are multifaceted, and determining their merits or worth is not evident seen multitudes of perspectives under which the interventions might be considered. Our study did not evaluate the *TITI* intervention as an operational study looking at the effectiveness or the operational merits of short-course TPT for young child contacts of TBB+ patients. As a matter of fact, both had been demonstrated already as early as in 2012 and were reconfirmed in a review and WHO´s recommendations in 2018 [10,11,17]. Our study explored the acceptability of the intervention by the targeted population and by the involved medical field staff and, then, confronting the results, evaluated the acceptability of the intervention intended as a means for demonstrating feasibility to those in charge of scaling it up. We might discern these two perspectives as ´intrinsic´ and ´extrinsic´ dimensions of acceptability.

The results of our study show that the intervention was largely welcomed and accepted by patients and field staff as long as executed in a project setting and in project mode, i.e. as long as the availability of resources and, thanks to them, a favourite economic, psycho-social climate to everybody involved was assured. ´Intrinsic´ acceptance of the intervention was possible because it evolved in a kind of vacuum, starting, in effect, with the administrative clearance at the central level for implementation and execution in a specific regional setting. The eagerness of the study´s patients and the willingness of the field staff to take part in the intervention are undoubtedly due to the extremely facilitating conditions in which the intervention took place, both for the patients and for the staff. Instead of being faced with all kinds of costs, opportunity costs included, access to services for parents of children has been facilitated in every possible way while the staff, instead of bearing costs, has rather been rewarded. On the other hand, patients´ and medical field staffs´ almost undivided enthusiasm about TB TPT prevention for children appears to be unexpected in view of the growing literature analysing reasons for reserves and practical obstacles among recipients and health personnel to scale up this intervention [5,17-19]. Thus, leaving the cocooned setting of the intervention, considerable scepticism and reservation characterized the attitude of the NTP management at regional and central level, including the medical field staff, when questioned about the feasibility and scaling-up perspectives under programme conditions. Financial and organizational feasibility are called into doubt outrightly, and when “additional activities” that the NTP cannot bear are evoked, the question of priorities and relevance of the intervention for the NTP´s policy arises.

The gap between the enthusiasm of the target populations and the actors directly involved in the intervention, on the one hand, and the stand-offish position expressed by the NTP actors responsible for management is unequivocal. It indicates the crux of the problem of an externally initiated what might be called a ‘showcase project` carried out in project mode by a development partner. The intervention implements what is internationally recommended, recognized as efficient, and pertinent. However, it lacks internal coherence with the current strategic priorities and practices of the national counterpart. Research and investment priorities of the NTP as well as its available resources are neglected. Thus, the intervention does not achieve its objective (change of practice) despite a considerable funder investment; the effectiveness and efficiency of the intervention as a promotional development intervention remain questionable, and the impact is doubtful.

The *TITI* intervention, conceived simultaneously as a showcase project and operational study, described here shares its fate with others, proposed in a proactive way, financed from outside, accepted and authorized administratively by a more or less isolated key player, and then planned for and organized by a handful field actors in a somewhat cocooned setting without questionable measurable impact. If there are advantages, gains, or benefits, they are foremostly short-term and destined for those directly concerned by and involved in the intervention during its execution, e.g. the participating children, the trained and financially motivated medical field staff, and, also hardly directly implicated, administrative facilitators. Finally, scientific capital is acquired by the initiators of the intervention and by those of the national counterpart implementers with whom this capital is shared [20].

**Limitations:** several limitations may have affected the results presented in this study. Firstly, there were many more actors concerned more indirectly by the intervention who were not interviewed for acceptability or appropriateness of the intervention for showing feasibility and promoting up-grading. Examples are colleagues of the medical staff involved, the radiologist hired during the project, the data manager or the NTP pharmacist or NTP management actors. They have been omitted from the assessment for convenience reasons. Data saturation had not been assured. Then, the methodological frameworks chosen and applied for the analysis of the data can be questioned. Describing, interpreting and evaluating merit and worth of the *TITI* study as a development intervention and its approach for demonstrating the feasibility and for encouraging acceptance had to fall back in a rather eclectic way on different normative frameworks, considering the complexity of the phenomena to assess. Thirdly, the coding and mapping of contributions collected during FGD and interviews were heavily influenced by the frameworks chosen. All three limitations did not allow to control entirely for interpretative biases. However, understanding materializes in a circle, unthinkable without some kind of previous conceptual structure and preunderstandings of the phenomenon, likely to shape the data collection, analysis, and interpretation. The method section of our study tried to make explicit the strategies employed to provide the backbone for our analysis, referred sometimes to as “axial coding” [21]. Finally, the study considers itself as a contribution to an ongoing discussion where over-accentuating of aspects of a complex phenomenon are unavoidable.

In general, assuring acceptability, demonstrating feasibility, and, thus, promoting acceptance under “real-life” conditions may look quite different than a model of care in project mode. It is under field conditions and with the all-out support and endorsement of the programme which has to be willing and able to make available the resources needed that an intervention has to be conceived and executed, owned then by the programme. As a critical retrospective of *TITI's* intervention in Benin states rightly: “further work remains to be done to better understand how to reach all children under five at a lower cost while ensuring reliable screening before starting TPT” [22].

## Conclusion

Generalized TPT with 3RH for children under five in contact with a bacilliferous TB patient is in principle accepted in Cameroon. A voluntaristic approach for promoting implementation, however rational and well-meant it may be, must take in account at least two conditions: the NTP must be closely associated to and involved in each stage of the planning, organization and M&E process; and real country ownership is needed, and considerable resources must be made available to introduce and maintain the strategy, resources to make the intervention patient-friendly, resources for running costs and incentives of the health staff and, last but not least, resources for the diagnostic means and the medications.

### What is known about this topic

- *Conflicting interests between the political interests of funders and their development initiatives and beneficiaries have become an issue discussed under the heading of country ownership or, more recently, decolonization, the latter notion without a generally accepted clear definition; the influence of colonial thinking on public health knowledge and policy-making is often rather spelled out by presenting and discussing examples;*
- *Few examples are present or discuss the instrumentalization heedless or with vested unspoken interests of local public health elites for the pursuit of policy implementation not necessarily in the interest of the concerned country*.

### What this study adds

- *This study, more exemplifying and exploring than systematically analysing, describes a situation typical for numerous donor interventions; funding and technical assistance;*
- *The introduction of a major disease control strategy is proposed to and accepted at central programme level; interviews of concerned actors, however, reveal an apparently successful funder intervention being neither organizationally nor financially feasible nor sustainable*.

## Annex

**Annex 1**: synopsis of participants´ (parents´) contributions during focus group discussions exploring the acceptability of tuberculosis preventive treatment for children aged less than 5 years old in selected health facilities in Douala, Cameroon (2023) (PDF - 87 KB)
